# Analysis of risk factors of hepatocellular carcinoma and establishment of a clinical prognosis model

**DOI:** 10.3389/fonc.2023.1067353

**Published:** 2023-03-22

**Authors:** Xin-Yu Ge, Ming-Chen Sun, Tian-Yi Wang, Xi-Min Wang, Gang Liu, Tao Yang, Yi-Ming Lu, Wei Wang

**Affiliations:** ^1^ First Affiliated Hospital of Jinzhou Medical University, Jinzhou, China; ^2^ Jinzhou Medical University, Jinzhou, Liaoning, China

**Keywords:** HCC (hepatic cellular carcinoma), SEER (Surveillance Epidemiology and End Results) database, machine learning - ML, risk factors, random survival forest model

## Abstract

Liver cancer is a common malignancy of the digestive system. Hepatocellular carcinoma (HCC) accounts for the most majority of these tumors and it has brought a heavy medical burden to underdeveloped countries and regions. Many factors affect the prognosis of HCC patients, however, there is no specific statistical model to predict the survival time of clinical patients. This study derived a risk factor signature of HCC and reliable clinical prediction model by statistically analyzing The Surveillance, Epidemiology, and End Results (SEER) database patient information using an open source package in the python environment.

## 1 Background

Liver cancer is a common malignancy of the digestive system (1, 2). Primary liver cancer mainly includes hepatocellular carcinoma (HCC) and intrahepatic cholangiocarcinoma (ICC) (3). HCC accounts for most of these tumors and is the fifth leading cause of cancer and the fourth leading cause of cancer-related deaths worldwide (4, 5).Men have a higher risk of HCC than women, comprising the second leading cause of cancer death in men. Besides, HCC morbidity and mortality are still rising (6, 7). The main risk factors for HCC development are cirrhosis and chronic liver disease (8). Cirrhosis is an important process for HCC viral carcinogenesis (9). Additionally, chronic hepatitis, caused by hepatitis B virus (HBV) and hepatitis C virus (HCV) infections, is an important risk factor for liver cancer (10). Most new liver cancer cases occur in developing countries with a high rate of hepatitis B virus infections. Meanwhile, non-alcoholic fatty liver disease (NAFLD) is the leading cause of HCC in developed countries (11, 12).

Liver Doppler ultrasound and AFP are simple and easy methods to screen liver cancer (13). Elevated AFP and DCP levels are typical features of liver cancer (14). Additionally, CT, enhanced CT, MRI, enhanced MRI, and other imaging methods are helpful for precise HCC diagnosis (15). Since liver biopsy is related to tumor implantation and bleeding risks, and false negative results might occur, it is generally not recommended for HCC (16).

At present, the most commonly used staging systems for liver cancer include the TNM (tumor node metastasis), China liver cancer (CNLC), and Barcelona clinical liver cancer (BCLC) staging systems (17). The TNM staging was jointly proposed by the American Joint Committee on Cancer (AJCC) and the Union for International Cancer Control (UICC) and has been widely used in clinical practice. TNM is a tumor staging system based on tumor morphology (T), regional lymph node metastasis (N), and distant metastasis (M). The TNM staging of liver cancer is very detailed, especially the T staging, including the invasion of microvessels around the tumor that can better help evaluate the prognosis.

Radical surgical resection is the primary treatment for early HCC. However, whether advanced HCC patients can benefit from surgery is controversial. Recently, breakthroughs have been made in non-surgical treatments. For example, drug therapy, immunotherapy, and targeted therapy have been successfully applied to treat advanced liver cancer (18). Transcatheter arterial chemoembolization (TACE), hepatic arterial infusion chemotherapy (HAIC), and radiotherapy can improve patient prognosis (19). Some experts believe that conventional chemotherapy can also benefit HCC patients (20). Nevertheless, most experts believe that conventional chemotherapy has little effect on liver cancer (21–23).

The SEER database is a publicly available cancer reporting system funded by the US federal government (24). This representative and reliable data come from 18 US states. Users can retrieve the patient’s sex, age, surgical method, chemotherapy, radiotherapy, other clinical information, survival time, and status. This study obtained permission to use the SEER PLUS database. Thus, to further explore HCC risk factors and treatment plans and establish a machine learning model to guide clinical treatment, we retrieved HCC patient data from the SEER database and analyzed them after the screening.

## 2 Methods

### 2.1 Data acquisition

Herein, we retrieved data from 107148 HCC patients from the SEER database. Clinical information included gender, age, race, histological type, histological grading, surgical method, regional lymph node dissection, radiotherapy, chemotherapy, diagnosis to treatment time, AFP, TNM staging, survival time, and survival status.

### 2.2 Excluding factors

To ensure the accuracy of the machine learning model, we did not use automatic imputation of missing information. Data were filtered according to the clinical characteristics of each group, and the information gaps and unknown groups were excluded from a total of 102680 patients. Finally, 4468 patients were selected for subsequent analysis.

### 2.3 Statistical methods

The algorithm applied here was based on python 3.10.6 (Python Software Foundation, https://www.python.org/). Clinical feature analysis was conducted with TableOne. The COX regression analysis was performed using Lifelines. The random survival forest (RSF) analysis was carried out using Scikit-Survival. The survival curves of clinical patients were predicted using the random forest model. The accuracy of the model was evaluated using the C-index.

## 3 Results

### 3.1 Clinical characteristics

After the screening, 4468 patients were selected for further analysis (**Table 1**) . The clinical characteristics were analyzed in **Table 1**. A total of 2324 patients received chemotherapy, and 2144 patients did not. Most clinical features significantly differed between the two groups, including gender, race, histological type, surgery, regional lymph node dissection, diagnosis to treatment time, survival time, AFP, survival status, and T, N, M stages (χ^2^ test, *p* < 0.05).

**Table 1:** Clinical characteristics.

		Grouped by Chemotherapy					
		Overall	0	1	P-Value	Test	SMD (0,1)
**n**		4468	2144	2324			
**Sex, n (%)**	**Female**	1031 (23.1)	568 (26.5)	463 (19.9)	<0.001	Chi-squared	0.156
	**Male**	3437 (76.9)	1576 (73.5)	1861 (80.1)			
**Race, n (%)**	**American Indian/Alaska**	60 (1.3)	28 (1.3)	32 (1.4)	<0.001	Chi-squared	0.214
	**Asian/Pacific**	906 (20.3)	530 (24.7)	376 (16.2)			
	**Black**	543 (12.2)	252 (11.8)	291 (12.5)			
	**White**	2959 (66.2)	1334 (62.2)	1625 (69.9)			
**Histological grade, n (%)**	**I**	1311 (29.3)	596 (27.8)	715 (30.8)	<0.001	Chi-squared	0.132
	**II**	2164 (48.4)	1111 (51.8)	1053 (45.3)			
	**III**	932 (20.9)	410 (19.1)	522 (22.5)			
	**IV**	61 (1.4)	27 (1.3)	34 (1.5)			
**Surgery, n (%)**	**None**	1816 (40.6)	214 (10.0)	1602 (68.9)	<0.001	Chi-squared (warning: expected count < 5)	nan
	**Local tumor destruction**	613 (13.7)	401 (18.7)	212 (9.1)			
	**Wedge or segmental resection**	813 (18.2)	702 (32.7)	111 (4.8)			
	**Lobectomy**	457 (10.2)	376 (17.5)	81 (3.5)			
	**Extended lobectomy**	122 (2.7)	89 (4.2)	33 (1.4)			
	**Hepatectomy**	633 (14.2)	352 (16.4)	281 (12.1)			
	**Excision of a bile duct**	1 (0.0)	1 (0.0)				
	**Excision of a bile duct & partial hepatectomy**	5 (0.1)	4 (0.2)	1 (0.0)			
	**Bile duct and hepatectomy WITH transplant**	8 (0.2)	5 (0.2)	3 (0.1)			
**Regional lymph node dissection, n (%)**	**None**	4055 (90.8)	1880 (87.7)	2175 (93.6)	<0.001	Chi-squared	0.204
	**1-3**	364 (8.1)	231 (10.8)	133 (5.7)			
	**≥**4	49 (1.1)	33 (1.5)	16 (0.7)			
**Radiation, n (%)**	**None**	3994 (89.4)	1924 (89.7)	2070 (89.1)	0.499	Chi-squared	0.022
	**Yes**	474 (10.6)	220 (10.3)	254 (10.9)			
**Time from diagnosis to treatment(months), median [Q1,Q3]**		2.0 [1.0,3.0]	1.0 [0.0,3.0]	2.0 [1.0,3.0]	<0.001	Kruskal-Wallis	0.033
**Survival time(months), median [Q1,Q3]**		33.0 [10.0,66.0]	54.0 [21.0,76.2]	18.0 [7.0,50.2]	<0.001	Kruskal-Wallis	-0.645
**AFP, n (%)**	**Negative**	1462 (32.7)	848 (39.6)	614 (26.4)	<0.001	Chi-squared	0.282
	**Positive**	3006 (67.3)	1296 (60.4)	1710 (73.6)			
**Survival status,n (%)**	**Alive**	1712 (38.3)	1168 (54.5)	544 (23.4)	<0.001	Chi-squared	0.672
	**Dead**	2756 (61.7)	976 (45.5)	1780 (76.6)			
**TNM-T, n (%)**	**T1**	2107 (47.2)	1260 (58.8)	847 (36.4)	<0.001	Chi-squared (warning: expected count < 5)	nan
	**T2**	1159 (25.9)	564 (26.3)	595 (25.6)			
	**T3a**	647 (14.5)	176 (8.2)	471 (20.3)			
	**T3b**	374 (8.4)	81 (3.8)	293 (12.6)			
	**T3NOS**	8 (0.2)	3 (0.1)	5 (0.2)			
	**T4**	171 (3.8)	60 (2.8)	111 (4.8)			
	**T0**	2 (0.0)		2 (0.1)			
**TNM-N, n (%)**	**N0**	4205 (94.1)	2118 (98.8)	2087 (89.8)	<0.001	Chi-squared	0.395
	**N1**	263 (5.9)	26 (1.2)	237 (10.2)			
**TNM-M, n (%)**	**M0**	4090 (91.5)	2084 (97.2)	2006 (86.3)	<0.001	Chi-squared	0.404
	**M1**	378 (8.5)	60 (2.8)	318 (13.7)			
**Age, median [Q1,Q3]**		62.0 [56.0,69.0]	62.0 [56.0,69.0]	62.0 [56.0,69.0]	0.479	Kruskal-Wallis	0.043

### 3.2 Overall risk factors

**Figure 1:**
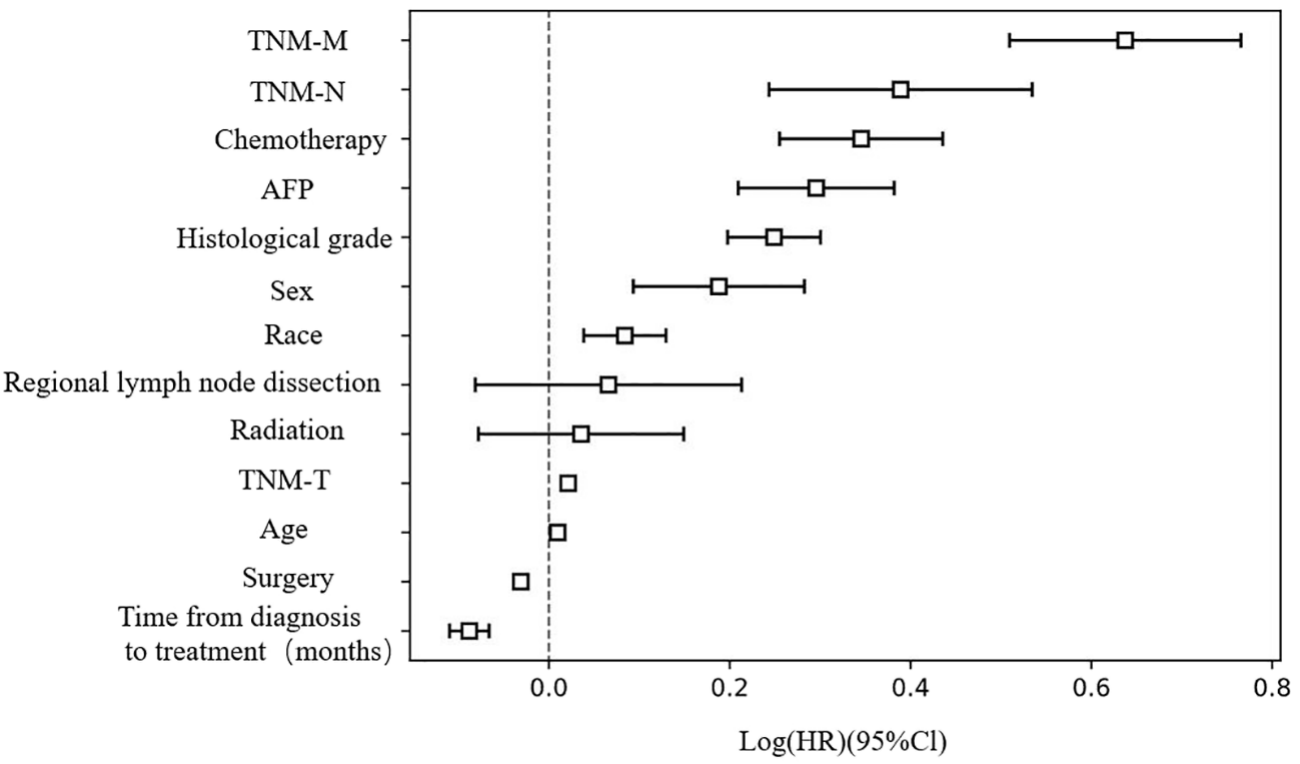
Risk factors assessment for HCC patients..

Furthermore, we used COX regression analysis to evaluate the impact of various clinical features on the survival of HCC patients (**Table 2**). Distant organ metastasis, lymph node metastasis, chemotherapy, AFP positive, histological grade, sex, race, tumor size, and age were risk factors for HCC. On the other hand, surgical treatment and early diagnosis and treatment were remission factors for HCC (*p* < 0.05). No significant differences were detected for radiotherapy and regional lymph node dissection (*p* > 0.05). The C-index of the COX regression model was 0.76 (**Figure 1**).

**Table 2:** Risk factors for survival.

	coef	exp(coef)	se(coef)	coef lower 95%	coef upper 95%	exp(coef) lower 95%	exp(coef) upper 95%	p	-log2(p)
**Sex**	0.19	1.21	0.05	0.09	0.28	1.1	1.33	<0.005	13.31
**Race**	0.08	1.09	0.02	0.04	0.13	1.04	1.14	<0.005	11.74
**Histological grade**	0.25	1.28	0.03	0.2	0.3	1.22	1.35	<0.005	68.75
**Surgery**	-0.03	0.97	0	-0.03	-0.03	0.97	0.97	<0.005	296.84
**Regional lymph node dissection**	0.07	1.07	0.08	-0.08	0.21	0.92	1.24	0.38	1.39
**Radiation**	0.04	1.04	0.06	-0.08	0.15	0.93	1.16	0.54	0.89
**Chemotherapy**	0.35	1.41	0.05	0.26	0.44	1.29	1.55	<0.005	43.84
**Time from diagnosis to treatment(months)**	-0.09	0.92	0.01	-0.11	-0.07	0.9	0.94	<0.005	48.23
**AFP**	0.3	1.34	0.04	0.21	0.38	1.23	1.47	<0.005	35.68
**TNM-T**	0.02	1.02	0	0.02	0.02	1.02	1.02	<0.005	150.79
**TNM-N**	0.39	1.48	0.07	0.24	0.53	1.28	1.71	<0.005	22.56
**TNM-M**	0.64	1.89	0.07	0.51	0.77	1.66	2.15	<0.005	72.38
**Age**	0.01	1.01	0	0.01	0.01	1.01	1.01	<0.005	22.23
Concordance	0.76								
Partial AIC	41495.47								
log-likelihood ratio test	2229.19 on 13 df								
-log2(p) of ll-ratio test	inf								

### 3.3 Risk factors at different stages

To explore the differences in treatment plans for HCC patients at different TNM stages, we divided patients into I, II, IIIa, IIIb, IVa, and IVb groups according to the 7th edition of the AJCC staging system. Then, we applied COX regression analysis to evaluate the risk for each group (**Table 3**). We found that early diagnosis and treatment, and timely surgery were mitigating factors for HCC patients at stages I, II, and IIIa. In contrast, chemotherapy, radiotherapy, and positive AFP were risk factors for HCC patients, unfavorable for prognoses. Surgical treatment and early diagnosis and treatment were also remission factors for stage IV HCC patients. Nevertheless, the prognosis risk was reduced in patients at stage IVa receiving radiotherapy, comprehending a mitigating factor. The survival of patients receiving chemotherapy did not differ. However, radiotherapy and chemotherapy were mitigating factors in the IVb group.

**Table 3:** Risk factors at different stages.

	I		II		IIIa		IIIb		IVa		IVb	
	exp(coef)	p	exp(coef)	p	exp(coef)	p	exp(coef)	p	exp(coef)	p	exp(coef)	p
**Sex**		1.31	1.09	0.41	1.23	0.02	1.32	0.3	1.67	0.12	0.93	0.61
**Race**	1.15	<0.005	1.13	0.02	1.03	0.43	0.98	0.84	0.97	0.81	1.01	0.83
**Histological grade**	1.17	<0.005	1.34	<0.005	1.33	<0.005	1.17	0.25	1.3	0.02	1.18	0.02
**Surgery**	0.97	<0.005	0.97	<0.005	0.97	<0.005	0.99	0.1	0.97	<0.005	0.97	<0.005
**Regional lymph node dissection**	1.09	0.54	0.87	0.39	0.95	0.7	1.04	0.87	1.69	0.11	0.73	0.19
**Radiation**	1.32	0.02	1.38	0.02	0.87	0.11	1.41	0.29	0.59	0.04	0.67	<0.005
**Chemotherapy**	1.6	<0.005	1.35	<0.005	1.17	0.09	1.3	0.3	1.13	0.68	0.67	0.01
**Time from diagnosis to treatment(months)**	0.93	<0.005	0.92	<0.005	0.87	<0.005	0.88	0.21	0.73	<0.005	0.8	<0.005
**AFP**	1.23	<0.005	1.47	<0.005	1.39	<0.005	1.33	0.31	1.42	0.11	1.26	0.1
**Age**	1.02	<0.005	1.01	0.01	1	0.55	1	0.94	1.01	0.37	1	0.87

### 3.4 Clinical feature importance and survival prediction

We randomly selected 25% of the included test group data, and the remaining 75% was used as the training group data. To obtain the best model, the survival analysis of the post-screening data was performed using the RSF model based on hyperparameter optimization with manual parameter adjustment, leading to a C-index of 0.80 for the training set and 0.77 for the testing set. Thus, the RSF model had slightly better reliability than the Cox regression model.

**Figure 2:**
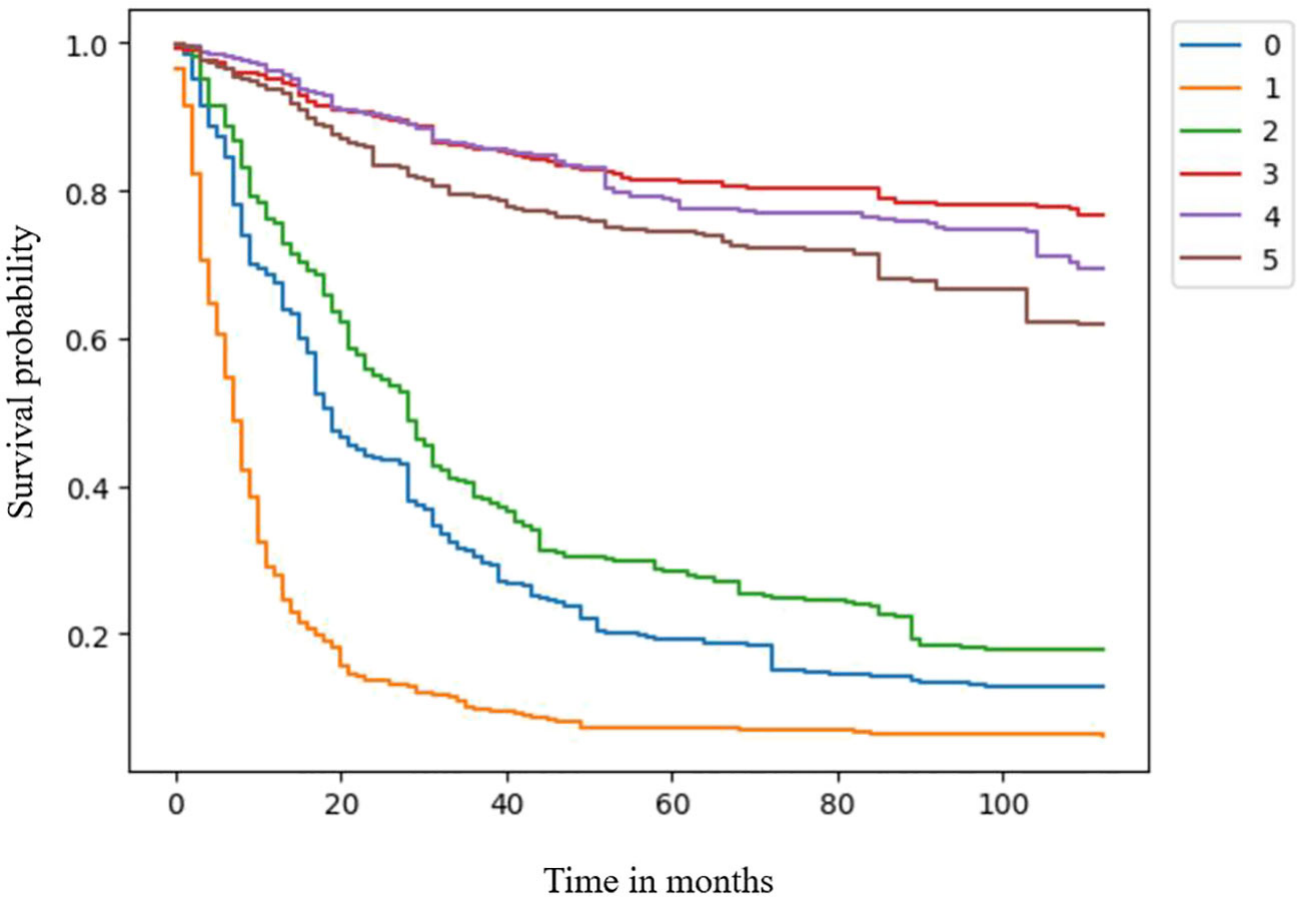
Survival curves for the surgery and non-surgery groups.

The clinical feature importance ranking indicated that surgical treatment was the most important feature among clinical factors in the RSF model (**Table 4**). Then, three patients in surgery and non-surgery groups were separately retrieved from the test group to draw predictive survival curves. Patients in the surgery group had a significantly better prognosis than those in the non-surgery group (**Figure 2**). 

**Table 4:** Clinical feature importance.

	Importances_mean	Importances_std
**Surgery**	0.109762	0.005483
**TNM-T**	0.036965	0.003964
**TNM-M**	0.011606	0.002241
**Histological grade**	0.007685	0.002583
**Time from diagnosis to treatment(months)**	0.006859	0.001441
**Age**	0.00568	0.001741
**TNM-N**	0.003543	0.000769
**AFP**	0.002642	0.001283
**Radiation**	0.001912	0.000937
**Race**	0.00102	0.001027
**Chemotherapy**	0.000678	0.001564
**Sex**	0.000492	0.00097
**Regional lymph node dissection**	0.000161	0.000466

Subsequently, we used Streamlit to establish a clinical patient survival prediction platform based on the RSF model. In this framework, clinicians can enter the corresponding clinical information, which is used to generate survival and cumulative risk curves of predicted patients and real-time survival curve changes by dynamically adjusting treatment parameters. Therefore, this platform can be used to guide clinical treatment selection (Video 1).

## 4 Discussion

The incidence and mortality of liver cancer continue to rise, and its treatment remains a global challenge (25). Surgery is the primary treatment of liver cancer (26). Nevertheless, liver cancer treatment has entered a new era with the development of immunotherapy and targeted therapeutic drugs. Since early liver cancer has no specific manifestation, few patients are diagnosed at early stages during regular physical examinations. Hence, most liver cancer patients are diagnosed at advanced stages when they present abdominal pain, jaundice, and other discomfort symptoms, missing the best time for treatment.

Moreover, HCC has brought a heavy medical burden to underdeveloped countries and regions (18).Chronic HBV infection, chronic HCV infection, NAFLD, aflatoxin, and alcohol intake are important causes of HCC. For example, Hepatitis B virus vaccination can reduce HCC incidence. Herein, the COX regression analysis showed that the time from diagnosis to treatment was a remission factor for HCC patients. Thus, early detection and timely treatment might improve the prognosis of HCC patients (HR: 0.92, *p* < 0.005). Thus, government departments and relevant medical security institutions should strengthen the health testing of high-risk HCC groups to achieve early detection and treatment, which can prolong the survival time of patients and reduce the economic burden on families and medical security institutions.

We found that positive AFP was also a risk factor for HCC patients at stages I, II, and IIIA. Hence, AFP can be used as an indicator of the prognosis of HCC patients, and similar conclusions have been reached in other studies (27).The Cox regression and RSF models indicated that surgery could reduce HCC risk and improve patient outcomes. Surgical treatment was the most important clinical feature affecting the survival of HCC patients in the RSF model, comprising a key factor for HCC management. For patients who can tolerate surgery, appropriate surgical treatment should be implemented as early as possible to avoid missing the optimal timing of treatment. Meanwhile, for patients not temporarily suitable for surgery, neoadjuvant treatments such as targeted therapy, immunotherapy, and TACE can be immediately implemented when the condition permits further operation.

We found that chemotherapy and radiotherapy were unsuitable for early liver cancer patients. Unnecessary radiotherapy and chemotherapy can increase the risk of these patients. However, chemotherapy can be used for advanced liver cancer patients, who might benefit from systemic chemotherapy (HR: 0.67, *p* < 0.005). Sun et al. showed that chemotherapy was a common treatment for advanced HCC, but the effects were not ideal. Adding all-trans-retinoic acid (ATRA) to fluorouracil, leuprorelin, and oxaliplatin (FOLFOX4) to treat advanced HCC can improve the overall survival and disease progression time of patients.

However, our study also has some limitations. First, the SEER database does not contain specific information on targeted therapy and immunotherapy regimens, which can extend the survival time of patients with recurrent or advanced liver malignancies. Second, we did not evaluate various objective factors affecting tumor patients’ survival time, such as economic conditions, medical insurance systems, and the level of medical development in the region. Finally, different machine learning models exhibit varying degrees of prognostic evaluation of patients. Therefore, this study should only be considered a machine-learning reference for treating tumor patients. With the continuous refinement of local databases and the optimization of artificial intelligence algorithms, machine learning models will be increasingly close to the reality of clinical practice.

Herein, we obtained a relatively reliable machine learning model by RSF. Then, we used this model to establish a survival prediction platform for HCC patients. This platform can generate a predicted survival curve by inputting clinical patient information. Survival curves can also be compared to get the best clinical treatment plan. Since the SEER database does not contain immunotherapy, targeted therapy, TACE, and other information, this platform only tests the feasibility of methods based on existing data to guide further research.

## 5 Conclusion

In the present study, we found that distant organ metastasis, lymph node metastasis, histological grade, sex, race, tumor size, and age were risk factors for HCC patients. Additionally, early detection and timely treatment might improve the prognosis of HCC patients, and positive AFP might be used as a risk indicator. Moreover, surgical treatment is crucial for HCC patient survival. Chemotherapy and radiotherapy are inappropriate for early liver cancer patients since these treatments can increase their risk. Nevertheless, advanced liver cancer patients might benefit from systemic chemotherapy. Finally, the RSF model can be used for clinical survival prediction.

## Data availability statement

The original contributions presented in the study are included in the article/**Supplementary Material**. Further inquiries can be directed to the corresponding author.

## Author contributions

X-YG is responsible for writing manuscripts and program codes; M-CS and T-YW are responsible for literature retrieval; X-MW, GL, Y-ML and TY are responsible for the program code; WW is responsible for proofreading and reviewing. All authors contributed to the article and approved the submitted version.
